# OmpW of *Caulobacter crescentus* Functions as an Outer Membrane Channel for Cations

**DOI:** 10.1371/journal.pone.0143557

**Published:** 2015-11-25

**Authors:** Roland Benz, Michael D. Jones, Farhan Younas, Elke Maier, Niraj Modi, Reinhard Mentele, Friedrich Lottspeich, Ulrich Kleinekathöfer, John Smit

**Affiliations:** 1 Department of Life Sciences and Chemistry, Jacobs University, Campus ring 1, 28759, Bremen, Germany; 2 Rudolf-Virchow-Center, DFG-Research Center for Experimental Biomedicine, University of Würzburg, 97078, Würzburg, Germany; 3 Department of Microbiology and Immunology, Life Sciences Centre, 2509–2350 Health Sciences Mall, University of British Columbia, Vancouver, British Columbia, V6T 1Z3, Canada; 4 Department of Physics and Earth Sciences, Jacobs University, Campus ring 1, 28759, Bremen, Germany; 5 Max-Planck Institute of Biochemistry, Department for Protein Analytics, Am Klopferspitz 18A, 82152, Martinsried, Germany; Centre National de la Recherche Scientifique, Aix-Marseille Université, FRANCE

## Abstract

*Caulobacter crescentus* is an oligotrophic bacterium that lives in dilute organic environments such as soil and freshwater. This bacterium represents an interesting model for cellular differentiation and regulation because daughter cells after division have different forms: one is motile while the other is non-motile and can adhere to surfaces. Interestingly, the known genome of *C*. *crescentus* does not contain genes predicted to code for outer membrane porins of the OmpF/C general diffusion type present in enteric bacteria or those coding for specific porins selective for classes of substrates. Instead, genes coding for 67 TonB-dependent outer membrane receptors have been identified, suggesting that active transport of specific nutrients may be the norm. Here, we report that high channel-forming activity was observed with crude outer membrane extracts of *C*. *crescentus* in lipid bilayer experiments, indicating that the outer membrane of *C*. *crescentus* contained an ion-permeable channel with a single-channel conductance of about 120 pS in 1M KCl. The channel-forming protein with an apparent molecular mass of about 20 kDa was purified to homogeneity. Partial protein sequencing of the protein indicated it was a member of the OmpW family of outer membrane proteins from Gram-negative bacteria. This channel was not observed in reconstitution experiments with crude outer membrane extracts of an OmpW deficient *C*. *crescentus* mutant. Biophysical analysis of the *C*. *crescentus* OmpW suggested that it has features that are special for general diffusion porins of Gram-negative outer membranes because it was not a wide aqueous channel. Furthermore, OmpW of *C*. *crescentus* seems to be different to known OmpW porins and has a preference for ions, in particular cations. A putative model for OmpW of *C*. *crescentus* was built on the basis of the known 3D-structures of OmpW of *Escherichia coli* and OprG of *Pseudomonas aeruginosa* using homology modeling. A comparison of the two known structures with the model of OmpW of *C*. *crescentus* suggested that it has a more hydrophilic interior and possibly a larger diameter.

## Introduction

The cell-envelope of Gram-negative bacteria consists of different layers. The inner or cytoplasmic membrane contains the respiration chain, proteins for the transport of nutrients and proteins involved in the synthesis of phospholipids, peptidoglycan and lipopolysaccharides [1, 2]. The periplasmic space between the membranes is an aqueous compartment iso-osmolar to the cytoplasm [3]. It contains the peptidoglycan and a large number of different proteins. The outer membrane is composed of protein, lipid and lipopolysaccharide (LPS) [1]. It typically contains only a few major proteins. Normally at least one of the constitutive outer membrane proteins is a porin, a general diffusion pore with a defined exclusion limit for hydrophilic solutes [4]. In addition to constitutive porins, an outer membrane may contain porins that are induced under special growth conditions [3]. They often form solute-specific channels and contain binding sites for neutral substrates such as carbohydrates [5, 6], or nucleosides [7] and phosphate [8, 9]. Many of the specific porins are part of uptake and degradation systems, such as the maltose uptake system of *Escherichia coli* [10].

*Caulobacter crescentus*, a member of the *alphaproteobacteria* group, is a Gram-negative bacterium found in oligotrophic aquatic environments [11]. *C*. *crescentus* has been studied as a model of cell cycle and bacterial differentiation. [12]. Its genome sequence has been known for more than 10 years [13]. *C*. *crescentus* is an unusual Gram-negative bacterium in that genes coding for typical general diffusion porins of the OmpF/C type of enteric gram-negative bacteria have not been identified in its genome [13–15]. Similarly, genes coding for specific porins such as Tsx or LamB are also absent. Instead, the genome of *C*. *crescentus* contains a large number of genes that code for TonB-dependent receptors [14, 16]. 67 of these receptors have been identified [15], which probably means that most of the nutrients from dilute environments are taken up actively by these systems. Examples for this are the uptake of maltose and N-acetyl glucosamine into the cells [15, 16].

In this study, we investigated whether the outer membrane of *C*. *crescentus* also contained a porin-like channel. The results suggested that despite the assumption that the *C*. *crescentus* outer membrane does not contain porins; a porin-like channel with a single-channel conductance of about 120 pS in 1 M KCl could be detected by adding crude outer membrane extracts to artificial membranes. The protein responsible for channel formation was identified to be a member of the large OmpW family of outer membrane proteins. OmpW homologues are found in many Gram-negative bacteria. Two members of this family, OmpW of *Escherichia coli* and OprG of *Pseudomonas aeruginosa* have been crystallized and their 3D-structures are known at high resolution (3.5 and 2.7 and 2.4 Å, respectively) [17–19]. Here we show that OmpW of *C*. *crescentus* functions as a channel for cations, which is in sharp contrast to OprG of *P*. *aeruginosa* and OmpW of *E*. *coli*, which are believed to be plugged or involved in the transport of small, yet unknown hydrophobic molecules across the cell wall of these bacteria [17–19]. The identity of OmpW of *C*. *crescentus* was verified by its deletion. A model for OmpW of *C*. *crescentus* was constructed on the basis of the known structures of OmpW of *E*. *coli* and OprG of *P*. *aeruginosa* [18, 19]. The results indicated that the OmpW channel of *C*. *crescentus* could have a larger diameter and a more hydrophilic interior than the two crystallized members of the OmpW family.

## Materials and Methods

### Growth and maintenance of microorganisms

*Caulobacter crescentus* CB15 NA1000 353Φ (JS1013) carries an amber mutation in *rsaA* resulting in S-layer deficiency [20]. This strain was used for initial identification and characterization of OmpW and for generation of an ompW-knockout strain. The strains were grown to mid-log phase (OD600 ~ 0.8) in peptone-yeast extract medium (PYE) [21] at 30°C in 5 ml cultures, which were used to start large cultures in 2.8 l Fernbach flasks containing 1250 ml M16HIGG medium, shaken at 100 rpm. M16HIGG is a modification of M6HIGG medium [22], containing 0.31% glucose, 0.09% glutamate, 1.25 mM sodium phosphate, 3.1 mM imidazole, 0.05% ammonium chloride and 0.5% modified Hutner’s Mineral Base.

### Outer membrane enriched preparations

Cells were pelleted by centrifugation at 12,400 *x g* for 10 min. Cell pellets were washed by suspension with distilled water and repelleted. The pellets were resuspended in 1/10 original culture volume of phosphate-buffered saline (PBS) [23] amended with 10 mM EDTA (PBS/EDTA), agitated at room temperature for 5 min and then centrifuged at 15,300 *x g* for 15 min. The supernatant was retrieved and re-centrifuged to clarify. The supernatant was then ultracentrifuged at 184,000 *x g* for 2 h. Glassy pellets formed which were suspended in 1/100 original culture volume in 10 mM Tris pH 8.0. This treatment led preferentially to the disruption of the outer membrane and periplasmic contents were released without significantly releasing cytoplasmic contents.

### Crude membrane preparations

For comparison to the PBS-EDTA membrane enrichment method, crude membrane preparations were prepared from 5 ml of mid logarithmic culture. The culture was sonicated at 50% intensity for 5 *x* 30 sec bursts. DNAse and RNAse were added to final concentrations of 0.06 mg/ml and 0.60 mg/ml, respectively, and incubated at 37°C for 1 h. The preparation was then ultracentrifuged for 2 h at 107,000 *x g*. A glassy pellet formed which was resuspended in 200 μl of distilled water.

### Generation of *C*. *crescentus* ΔompW

The gene *ompW*, CCNA_01475 (CC_1409), which encodes for OmpW, was knocked out in wildtype *C*. *crescentus* NA1000 via a two step method, derived from previously published protocols [20], resulting in a dysfunctional copy of *ompW* with a large internal deletion. The suicide plasmid ‘pKMOBsacB-ompW-A/B’ was constructed by combining two fragments of DNA homologous to the 5’ and 3’ ends of the gene *ompW* into a suicide vector containing both positive and negative selection elements. The ‘A-fragment’ was PCR amplified using the primers F-ompW-A (5’- TAC CGG AAT TCT CGG GCG CTG GGC CTG TCT GTT GAG -3’) and R-ompW-A (5’- GTC CCA AGC TTG CGG AAG ATC TAT TGG CGC CGG CGG CAG TCA GGA TG -3’), resulting in a 994 bp product with a 5’ *Eco*RI cleavage site and 3’ *Bgl*I and *Hin*DIII cleavage sites. The ‘A-fragment’ PCR product was blunt ligated into pBSK-ESH [24], resulting in pBSK-ompW-A. The ‘B-fragment’ PCR product was amplified using the primers F-ompW-B (5’- CGG GAT CCA CGT CAA GAA GGT CTA TTT CAG CAC -3’ and R-ompW-B (5’- GTC CCA AGC TTG CGT CGA TGC TAG TGC GCT GCG ATG -3’), resulting in a 1090 bp product with a 5’ *Bam*HI cleavage site and a 3’ *Hin*DIII cleavage site. The ‘B-fragment’ PCR product was similarly blunt subcloned into pBSK-ESH, then excised by *Bam*HI and *Hin*DIII digestion, and ligated into pBSK-ompW-A, digested with *Bgl*II and *Hin*DIII, resulting in the plasmid pBSK-ompW-A/B. The plasmids pBSK-ompW-A/B and pKMOBsacB were digested with *Eco*RI and *Hin*DIII, the 2070 bp fragment from pBSK-ompW-A/B was ligated into the pKMOBsacB fragment creating the plasmid pKMOBsacB-ompW-A/B.

The plasmid pKMOBsacB-ompW-A/B was electroporated into *C*. *crescentus* NA1000 cells [25]. The resulting transformants that were kanamycin resistant were re-passaged through kanamycin-free PYE medium three times and plated on kanamycin-free PYE agar plates that contained 3% sucrose. All colonies that were found to be sucrose resistant were screened for kanamycin sensitivity to confirm that the pKMOBsacB-ompW-A/B plasmid had crossed out of the genome. Colonies that were both sucrose resistant and kanamycin sensitive had their *ompW* genes PCR amplified using the primers F-ompW (5’- CGC ACT GGG CTT GCT GGC CTT TTT C -‘3) and R-ompW (5’- GGA GCC AGA GGA CGG ACG ACC GGG G -‘3); intact *ompW* resulted in a roughly 800 bp product, and knocked-out *ompW* resulted in a roughly 500 bp product.

### Isolation and purification of the channel-forming protein from enriched outer membranes

The enriched outer membranes prepared by PBS/EDTA extraction were inspected for channel-forming activity by treatment with the detergent lauryldimethylamine-oxide (LDAO). The detergent extracts of the enriched OM showed rapid channel formation in the lipid bilayer assay. The protein responsible for channel formation was identified by preparative SDS-PAGE. Highest channel-forming activity was observed in the molecular mass range between 20 and 25 kDa.

### SDS-PAGE

Analytical and preparative SDS-PAGE was performed according to [26]. The gels were stained with Coomassie brilliant blue or colloidal Coomassie blue [27]

### Tryptic digestion and peptide sequencing

The pure 22 kDa protein eluted from preparative SDS-PAGE was subjected to amino acid sequence analysis using an ABI 472A protein sequencer (Applied Biosystems, Langen, Germany). Direct sequencing was not successful, presumably because of the blocking of the N-terminus. The 22 kDa protein was therefore cleaved with trypsin as described by Eckerskorn and Lottspeich (1989) [28]. The peptides were separated by reversed phase HPLC on a Purospher RP18 encapped 5 μm column (Merck, Darmstadt, Germany) using a solvent gradient from 0 to 60% acetonitrile in 0.1% trifluoroacetic acid/water (v/v). The flow rate was 60 μl/minute and UV-detection was performed at 206 nm. The amino acid sequences of the tryptic peptides were analysed using Mass spectrometry.

### Lipid bilayer experiments

The method used for the reconstitution experiments using black lipid-bilayer membranes has been described previously [29]. The membranes were formed from a 1% (w/v) solution of diphytanoyl phosphatidylcholine (PC) (Avanti Polar Lipids, Alabaster, AL, U.S.A.) in n-decane. The membrane current was measured with a pair of calomel electrodes switched in series with a voltage source and an electrometer (Keithley 617). For single-channel recordings, the electrometer was replaced by a highly sensitive current amplifier (Keithley 427). Zero-current membrane potentials were measured in the following way [30, 31]: the membranes were formed in 0.1 M salt solutions. Porin was added to both sides of the membrane. After reconstitution of 100 to 1000 channels in the membrane a salt gradient was established across the membranes by addition of small amounts of concentrated salt solution to one side of the membrane and the same volume of 0.1 M salt solution to the other while stirring both compartments. The resulting zero-current membrane potentials were measured about 5 minutes after the salt gradients were established using a high impedance electrometer (Keithley 617).

### Construction of the OmpW model

The model of OmpW of *C*. *crescentus* was derived using the homology modeling approach. A three dimensional model of *C*. *crescentus* OmpW was built using the Modeller program [32], taking *E*. *coli* OmpW as a template structure [18].

## Results

### Protein composition of the enriched outer membranes of *C*. *crescentus*

**Fig 1:**
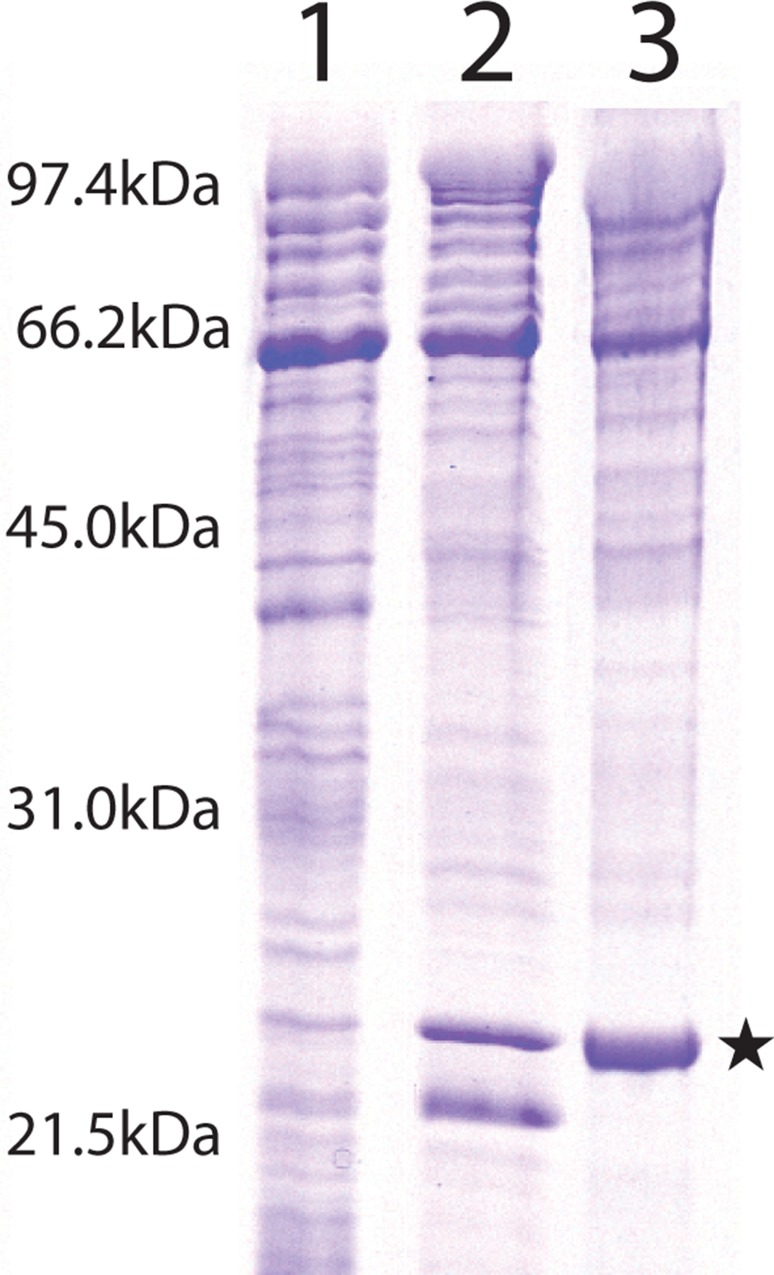
Coomassie stained, 12% SDS-PAGE of different protein preparations of *C*. *crescentus*. Lane 1, Crude membrane preparation. Lane 2, PBS-EDTA extract, not boiled prior to loading. Lane 3, PBS-EDTA extract, boiled prior to loading. The star highlights the position of OmpW.

Crude membranes from *C*. *crescentus* CB15A NA1000 353Φ cells that were disrupted by sonication and membrane material released by PBS/EDTA treatment were analyzed by SDS-PAGE. The membranes from the sonicated cells (presumably a mixture of the cytoplasmic and outer membranes) (Fig 1, lane 1) and the membranes from the PBS/EDTA extract (Fig 1, lanes 2 and 3) showed numerous proteins present. The PBS/EDTA extracted cell membranes showed some enrichment in outer membrane proteins (see Fig 1, lanes 2 and 3). The proteins of lane 2 (not boiled before analysis) showed an enrichment of two protein bands at about 20 and 22 kDa. When the PBS/EDTA extract preparations were boiled prior to SDS-PAGE, the two enriched bands ran as a single band of about 22 kDa in size (Fig 1, lane 3). This “heat modifiability” of the enriched protein (i.e., lower mobility when boiled in the presence of SDS) is often observed for outer membrane proteins of Gram-negative bacteria and is probably caused by their unfolding [33].

### Identification of channel-forming activity in enriched outer membranes of *C*. *crescentus*

**Fig 2:**
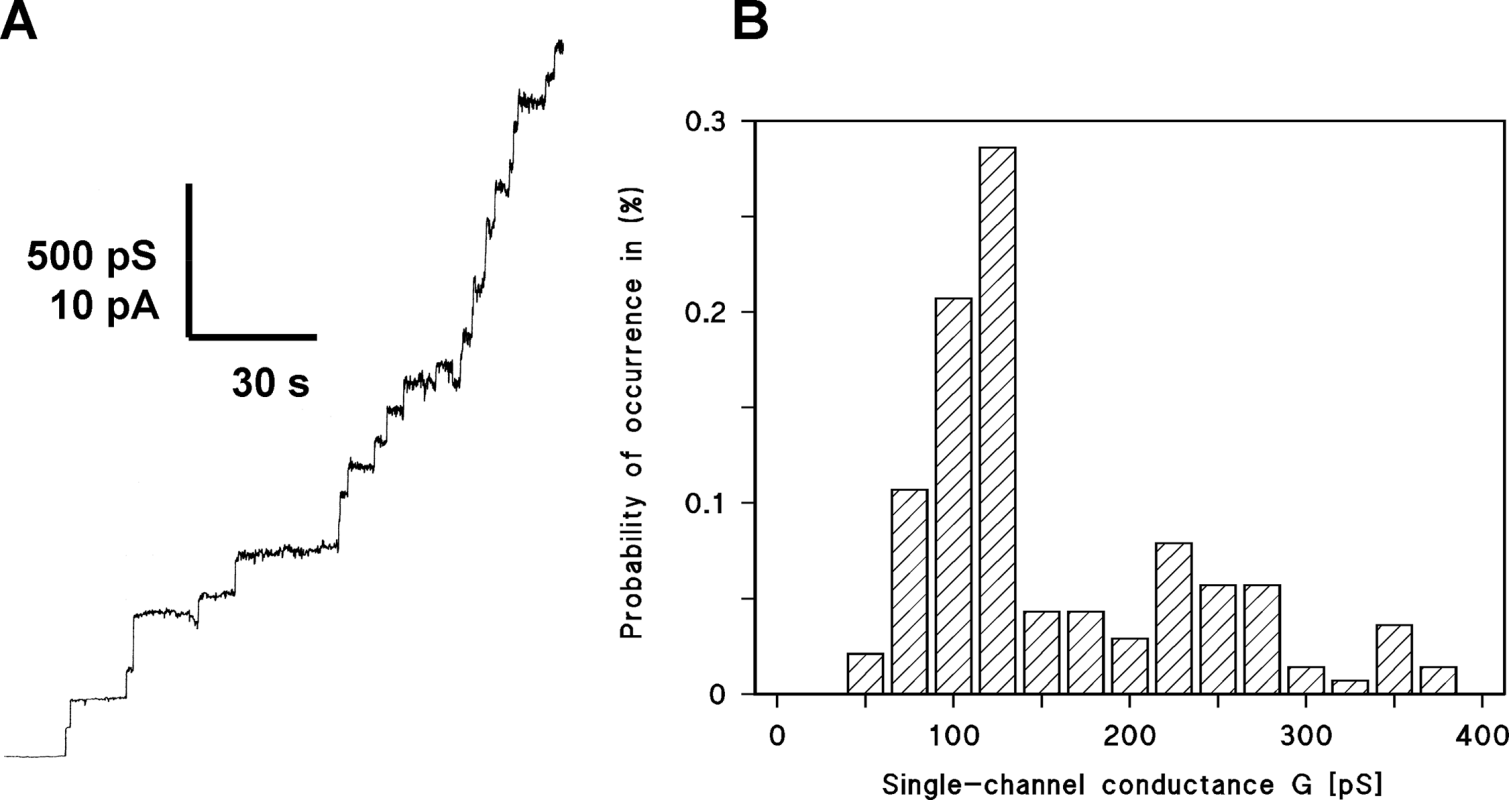
A. Single-channel recordings of a PC/*n*-decane membrane in the presence of enriched outer membranes of *C*. *crescentus*. The aqueous phase contained 1 M KCl and 100 ng ml^−1^ protein from enriched outer membranes treated with 0.5% LDAO. The applied membrane potential was 20 mV; *T* = 20°C. B. Histogram of the probability *P*(*G*) for the occurrence of a given conductivity unit observed with membranes formed of PC/*n*-decane in the presence of enriched outer membranes of *C*. *crescentus*. *P*(*G*) is the probability that a given conductance increment *G* is observed in the single-channel experiments. It was calculated by dividing the number of fluctuations with a given conductance increment by the total number of conductance fluctuations. The aqueous phase contained 1 M KCl. The applied membrane potential was 20 mV; T = 20°C. A Gaussian function was applied to the histogram and the maximum of the curve was taken as average single-channel conductance. It was 113 (± 22) pS for 105 single-channel events (left-hand maximum).

The enriched outer membranes were extracted with 0.5% LDAO. When small amounts of this detergent extract (containing about 5 μg of protein) were added to one or both sides of a black PC/n-decane membrane (volume 5 ml) we observed a significant increase of membrane conductance indicating that the enriched outer membranes contained components that had ion-conducting activity. The conductance increase was delayed after addition of the extract. After an initial rapid increase of conductance for 5 to 10 minutes, the increase slowed down and saturated at 30 to 60 minutes after addition of the detergent solution to the black membranes. When the detergent alone was added at the same concentration as with the PBS/EDTA extracted membranes it had no influence on the conductance of the lipid bilayer membranes. When the detergent extract of the outer membranes was added at much lower protein concentrations (about 500 ng protein) to the aqueous phase (5 ml volume) bathing the black lipid membrane, the current increased in a step-wise fashion (see Fig 2A). A histogram of the channel distribution demonstrated that most of the conductance steps had a conductance of about 120 pS in 1M KCl (see Fig 2B). Besides the 120 pS channel, some fluctuations with higher conductance (around 250 pS) were also observed which probably represented simultaneous reconstitution of several of the 120 pS channels possibly from porin micelles that could not be separated at the time scale of our experimental conditions. The conductance of these steps was definitely much lower than that of general diffusion pores from enteric bacteria, which is at least 2 to 4 nS for the trimers in 1 M KCl, corresponding to 700 to 1,300 pS per monomer [3]. This result suggested that the PBS/EDTA extracted membranes of *C*. *crescentus* contained a porin-like channel with small conductance.

### Identification of the channel-forming protein from the enriched outer membranes

**Fig 3:**
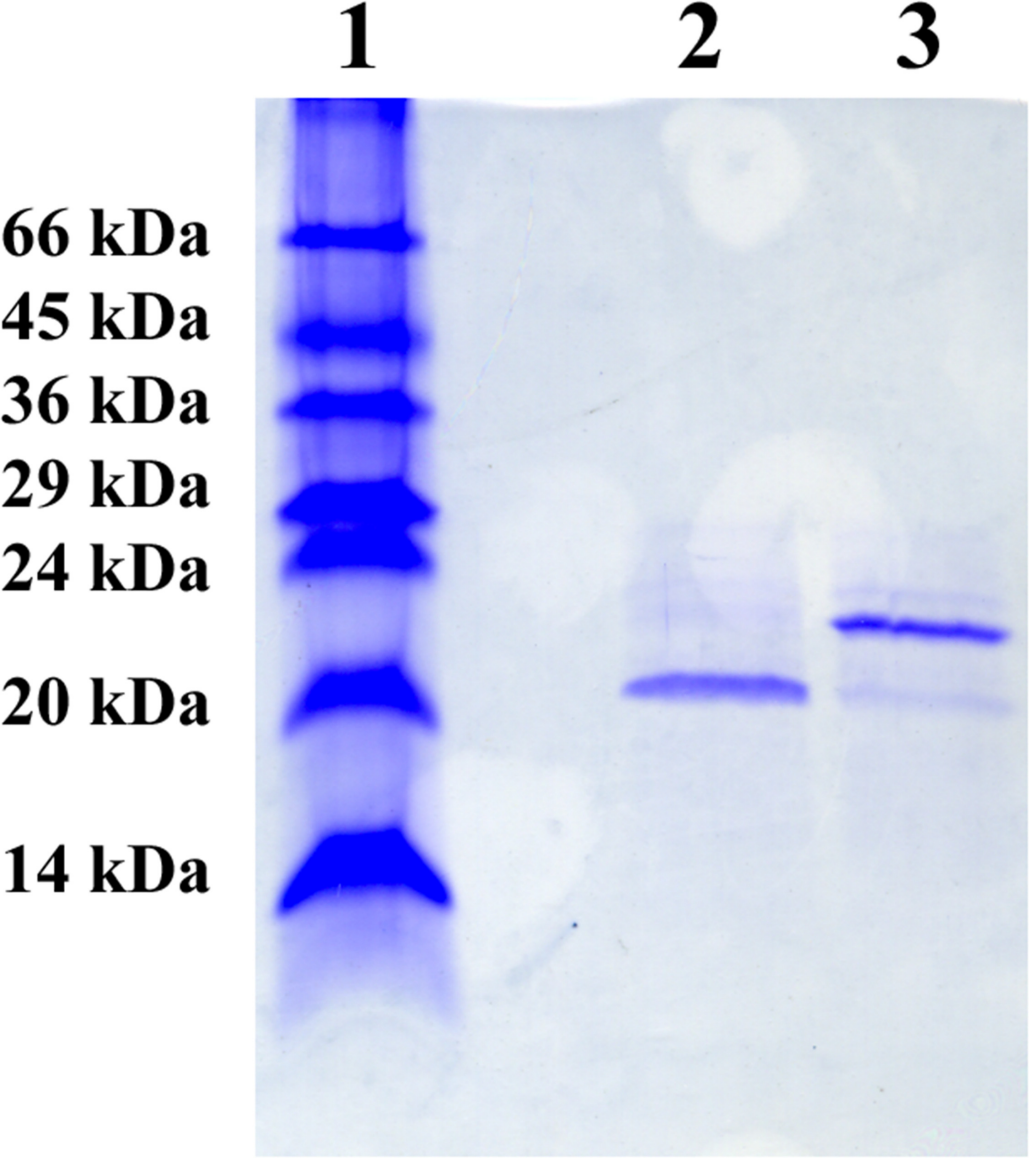
Coomassie stained, 15% SDS-PAGE of OmpW of *C*. *crescentus* obtained by elution of the 20 kDa band from preparative SDS-PAGE. Lane 1: Molecular mass. Lane 2: OmpW solubilized at room temperature for 10 min in 5μl sample buffer. Lane 3: OmpW solubilized at 100°C for 10 min in 5 μl sample buffer.

To identify the protein responsible for the channel-forming activity, the PBS/EDTA extracted membranes were dissolved in detergent and subjected to preparative SDS−PAGE. The gel was cut into thin slices, corresponding to defined molecular mass ranges, and each was eluted overnight with a buffer containing 1% of the detergent Genapol. The eluted molecular mass fractions were examined for channel-forming activity in the lipid bilayer assay. Extremely high channel-forming activity was exclusively localized within the molecular mass range 20 to 22 kDa, corresponding to the protein bands enriched by PBS/EDTA extraction (see Fig 1). The other bands from the gel, in particular the 66 kDa band, had no activity in the lipid bilayer assay. When excised and eluted, the band was again subjected to SDS-PAGE. Without heating, the gel showed a single protein band of about 20 kDa suggesting that the excised protein was essentially pure (Fig 3, lane 2). When the excised 20 kDa band was heated to 100°C prior to addition to SDS-PAGE, most of the protein ran at an apparent molecular mass of about 22 kDa (Fig 3, lane 3), with indication that some of the protein ran at 20 kDa (Fig 3, lane 3). This suggested again that the 20 kDa protein from *C*. *crescentus* is heat modifiable.

### Partial sequencing of the 20–22 kDa protein of *C*. *crescentus*

The 20 kDa protein was subjected to sequencing in a first run using Edman-degradation. However, the protein could not be sequenced starting from the N-terminus, presumably because of N-terminal blockage. Following trypsin treatment and separation of the resulting peptides, one prominent stretch with a molecular mass of 1764.8 could be resolved by mass spectrometry. Mascot search (http://www.matrixscience.com) was performed and the partial peptide QDFTPNAKGDLIVHAR was obtained. A NCBI BLAST search (Basic Local Alignment Search Tool) [34, 35] of the sequenced peptide unambiguously demonstrated that it was the N-terminal peptide of OmpW (CC_1409) of *C*. *crescentus*. Glutamine is the first amino acid of mature OmpW suggesting that the N-terminus was blocked by the formation of pyroglutamate. OmpW is a member of the extensive OmpW-family of outer membrane proteins of Gram-negative bacteria. To ensure that the higher molecular mass band observed in boiled samples was also OmpW; this protein band (about 22 kDa) was also subjected to mass spectrometry following trypsin treatment. Its N-terminal end was identical to that of the 20 kDa protein (OmpW), indicating again that OmpW is heat-modifiable, which led to two different conformations of the protein.

### Analysis of the channels formed by OmpW channel-forming protein of *C*. *crescentus*

**Fig 4:**
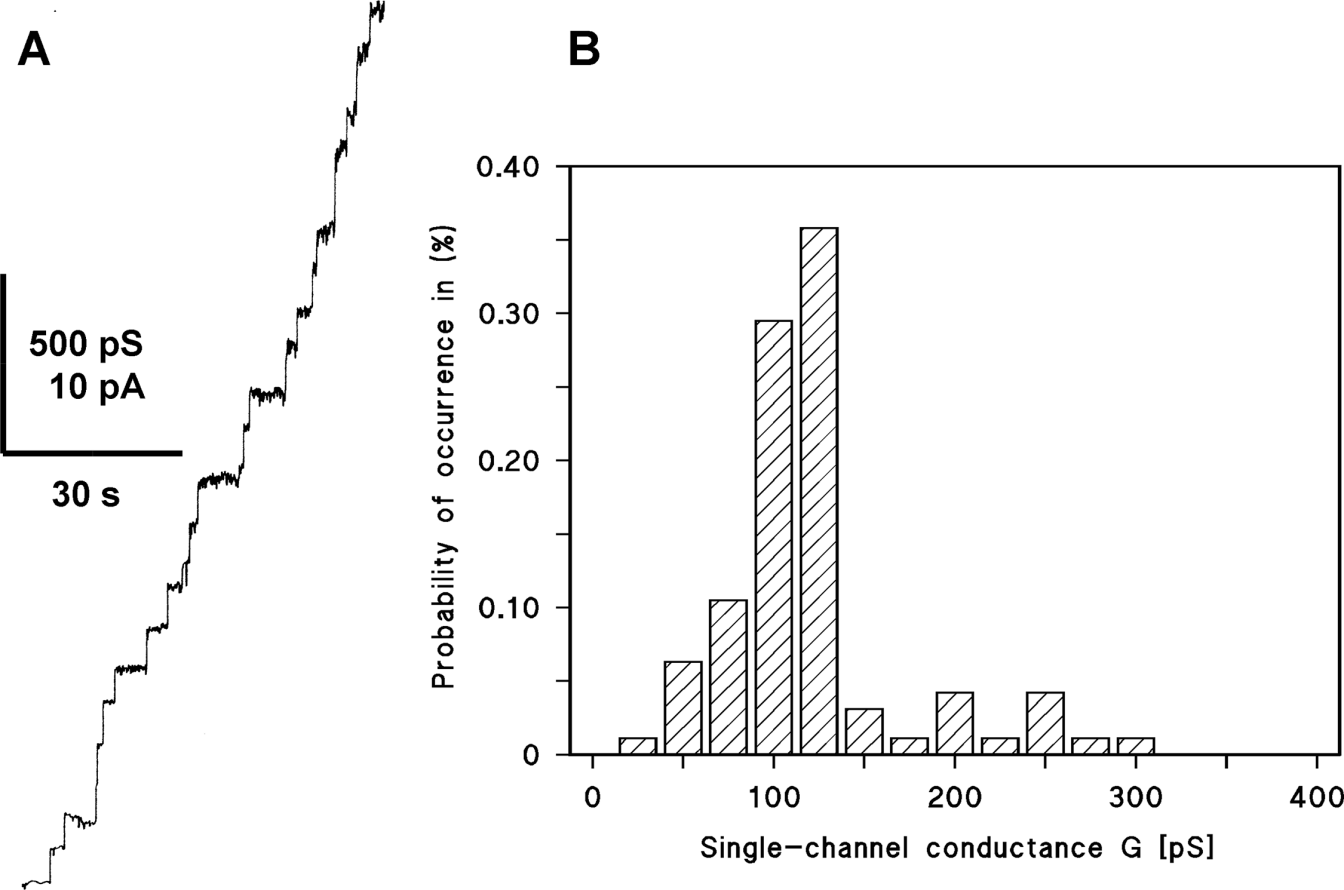
A. Single-channel recordings of a PC/*n*-decane membrane in the presence of purified OmpW of *C*. *crescentus*. The aqueous phase contained 1 M KCl and 20 ng ml^−1^ OmpW dissolved in 1% Genapol. The applied membrane potential was 20 mV; *T* = 20°C. B. Histogram of the probability *P*(*G*) for the occurrence of a given conductivity unit observed with membranes formed of PC/*n*-decane in the presence of OmpW of *C*. *crescentus*. *P*(*G*) is the probability that a given conductance increment *G* is observed in the single-channel experiments. It was calculated by dividing the number of fluctuations with a given conductance increment by the total number of conductance fluctuations. The aqueous phase contained 1 M KCl and about 20 ng/ml OmpW. The applied membrane potential was 20 mV; T = 20°C. A Gaussian function was applied to the histogram and the maximum of the curve was taken as average single-channel conductance. It was 117 (± 19) pS for 95 single-channel events.

Fig 4A shows a single-channel recording of a PC membrane in the presence of the purified OmpW protein of *C*. *crescentus*, which was added to a black lipid membrane at a concentration of about 20 ng/ml. The single-channel recording demonstrates that the protein formed the same defined channels as found in PBS/EDTA extracted membranes of *C*. *crescentus*. The average single-channel conductance was about 120 pS in 1 M KCl (almost 40% of all conductance steps) as derived from a fit of the single data by a Gaussian function. Only a minor fraction of channels with other conductance was observed (see the histogram in Fig 4B) suggesting that conductance steps with more than one unit conductance were less frequent for the purified OmpW than for the crude outer membrane fraction, which may contain more OmpW micelles. It is noteworthy that the channels formed by OmpW of *C*. *crescentus* had a long lifetime, similar to those that have been detected previously for porins of Gram-negative [3] and Gram-positive bacteria [36, 37]. All these channel-forming proteins from the cell walls of bacteria form channels in lipid bilayer membranes with long lifetimes at small transmembrane potential (mean lifetime at least 5 minutes). Furthermore, no voltage-dependence closure was observed in KCl-solution voltages up ±120 mV (data not shown).

Single-channel experiments were also performed with salts other than KCl to obtain information on the size and selectivity of the channels formed by OmpW of *C*. *crescentus*. The results are summarized in Table 1. The conductance sequence of the different salts within the channel was KCl ≈ KCH_3_COO ≈ NH_4_Cl > RbCl > NaCl > CsCl > LiCl. The influence of cations of different size and mobility on the conductance was substantial (see Table 1) suggesting indeed high cation-selectivity of the OmpW channel. We observed a very low conductance of much less than 10 pS for more bulky cations such as N(CH_3_)_4_^+^ or the Tris cation, suggesting that the size of the OmpW channel was indeed very small.

**Table 1:** Average single-channel conductance, G, of OmpW of *C*. *crescentus* in different salt solutions and radii, hydrated radii and limiting molar conductivity of the cations.

Salt	Concentration	Single-channel conductance G	Crystal ion radius r	Hydrated ion (Stokes) radius a	Limiting molar conductivity λi
	[M]	[pS]	[nm]	[nm]	[mS/M]
LiCl	1.0	15 ± 3.0	0.059	0.216	38.68
NaCl	1.0	40 ± 3.5	0.100	0.163	50.10
KCl	0.01	30 ± 3.2	0.137	0.110	73.50
	0.03	40 ± 3.4			
	0.1	55 ± 4.9			
	0.3	80 ± 6.2			
	1.0	117 ± 19			
	3.0	150 ± 8.9			
NH4Cl	1.0	125 ± 9.5	0.147	0.110	73.55
RbCl	1.0	100 ± 7.6	0.152	0.105	77.81
CsCl	1.0	30 ± 2.9	0.167	0.106	77.26
(CH_3_)_4_NCl	1.0	<10	0.347	0.182	44.92
KCH_3_COO^-^	0.1	60 ± 8.0			
pH 7	1.0	125 ± 10			
CaCl_2_	0.5	225 ± 22			
	1.0	250 ± 19			
MgCl_2_	1.0	275 ± 27			

The membranes were formed from diphytanoyl phosphatidylcholine dissolved to 1% in *n*-decane. The aqueous solutions were used unbuffered and had a pH of 6 unless otherwise indicated. The applied voltage was 20 mV, and the temperature was 20°C. The average single-channel conductance, G (± SD), was calculated from at least 80 single events by averaging over all fluctuations or using a Gaussian distribution of the single-channel conductance (1 M KCl). The ionic radii of the ions were taken from literature sources [38–40]. The radii of the hydrated cations were calculated from the limiting conductivities using the Stokes equation and were taken from [37]. The data for the limiting conductivities of the different ions were taken from [41]. The fact that the hydrated ion radius (Stokes radius) is in some cases smaller than the crystal radius is a phenomenon that is known from the literature (see ref. [42]).

Table 1 contains besides the results of the single channel measurements with OmpW of *C*. *crescentus* also the crystal ion radii of the different monovalent cations taken from different references for comparison [38–40]. Similarly, Table 1 shows also the hydrated ion (Stokes) radii (taken from ref. [37]) for comparison as calculated from the limiting molar conductivities of the cations [41] using the Stokes equation. The fact that, contrary to expectation, the hydrated ion radius (Stokes radius) is in some cases smaller than the crystal radius is a well-known phenomenon that is also known from the literature (see ref. [42]). It is presumably caused by some in theory neglected interaction of ions with the solvent (water) molecules [42].

An additional interesting result of the single-channel measurements was the extreme high conductance of OmpW in CaCl_2_ (see Table 1). In 1M CaCl_2_, the conductance was about 250 pS; this was again close to saturation because in 0.5M CaCl_2_ the conductance was only little lower than in a 1M solution. Interestingly, conductance traces in CaCl_2_ solutions were very noisy suggesting a strong interaction between the divalent cations and the OmpW channels. Similarly, channel-forming activity in salt solutions containing divalent cations was much lower than that in monovalent cation solutions, indicating fewer reconstitution events. This could mean that OmpW exhibits a special interaction with divalent cations, such as Ca^2+^ or Mg^2+^. Table 1 also shows the average single-channel conductance, G, as a function of the KCl concentration in the aqueous phase. Similarly, as in the case of some channels of Gram-positive bacteria [36,37,43] the conductance was not a linear function of the KCl-concentration, which means that OmpW did not form a wide, water-filled channel. The saturation with increasing salt concentration could be caused by point net charges and/or a binding site for ions.

### The OmpW channel of *C*. *crescentus* is highly cation-selective

Additional information about the properties of the channel formed by OmpW of *C*. *crescentus* was obtained from zero-current membrane potential measurements in presence of salt gradients. A fivefold KCl gradient (100 mM versus 500 mM), across a lipid bilayer membrane in which about 100 to 1000 OmpW channels were reconstituted, resulted in a zero-current membrane potential of 35 ± 3 mV at the more dilute side (mean of 3 measurements). This result indicated preferential movement of potassium ions over chloride through the channel at neutral pH. The zero-current membrane potentials were analyzed using the Goldman-Hodgkin-Katz equation [30, 31]. The ratio of the potassium permeability, PK, divided by the chloride permeability, PCl, was about 15 (mean of 3 measurements), indicating high cation selectivity of the channel formed by OmpW (see also Discussion). This result was confirmed by measurements with LiCl and potassium acetate; we observed under the same conditions as for KCl, zero-current membrane potentials around 32 to 35 mV for fivefold gradients (mean of three experiments), meaning that P_anion_/P_cation_ was also in these cases higher than 10. More precise numbers cannot be expected because small errors of the zero-current membrane potential in the range of 32 to 35 mV for fivefold gradients result in big variations of the permeability ratio P_anion_/P_cation_. Size and mobility of cations and anions did not influence the cation selectivity of OmpW in contrast to the situation observed previously for general diffusion porins of enteric bacteria [31].

### Investigation of enriched outer membranes of an OmpW-deficient *C*. *crescentus* strain for pore-forming activity

**Fig 5:**
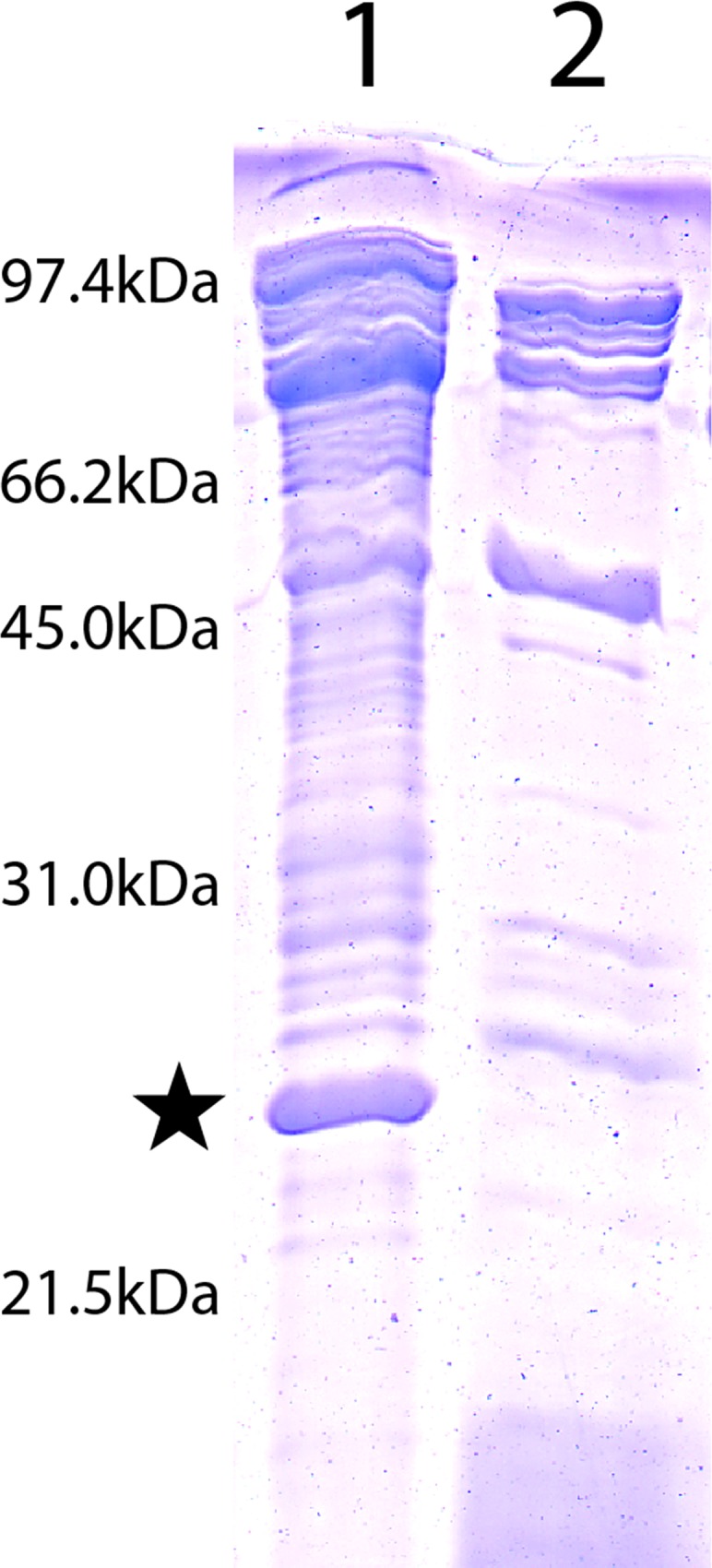
Coomassie stained, 12% SDS PAGE of PBS-EDTA extractions from wildtype and *C*. *crescentus* Δ*ompW*. Lane 1, *C*. *crescentus* PBS-EDTA extract. Lane 2, *C*. *crescentus* ΔompW PBS-EDTA extract. While PBS-EDTA extraction was a convenient method to enrich for OmpW, there was some variability between extractions, which can be seen in comparing protein profiles in lanes 1 and 2. Equal volume loadings were used. The star highlights the position of OmpW.

**Fig 6:**
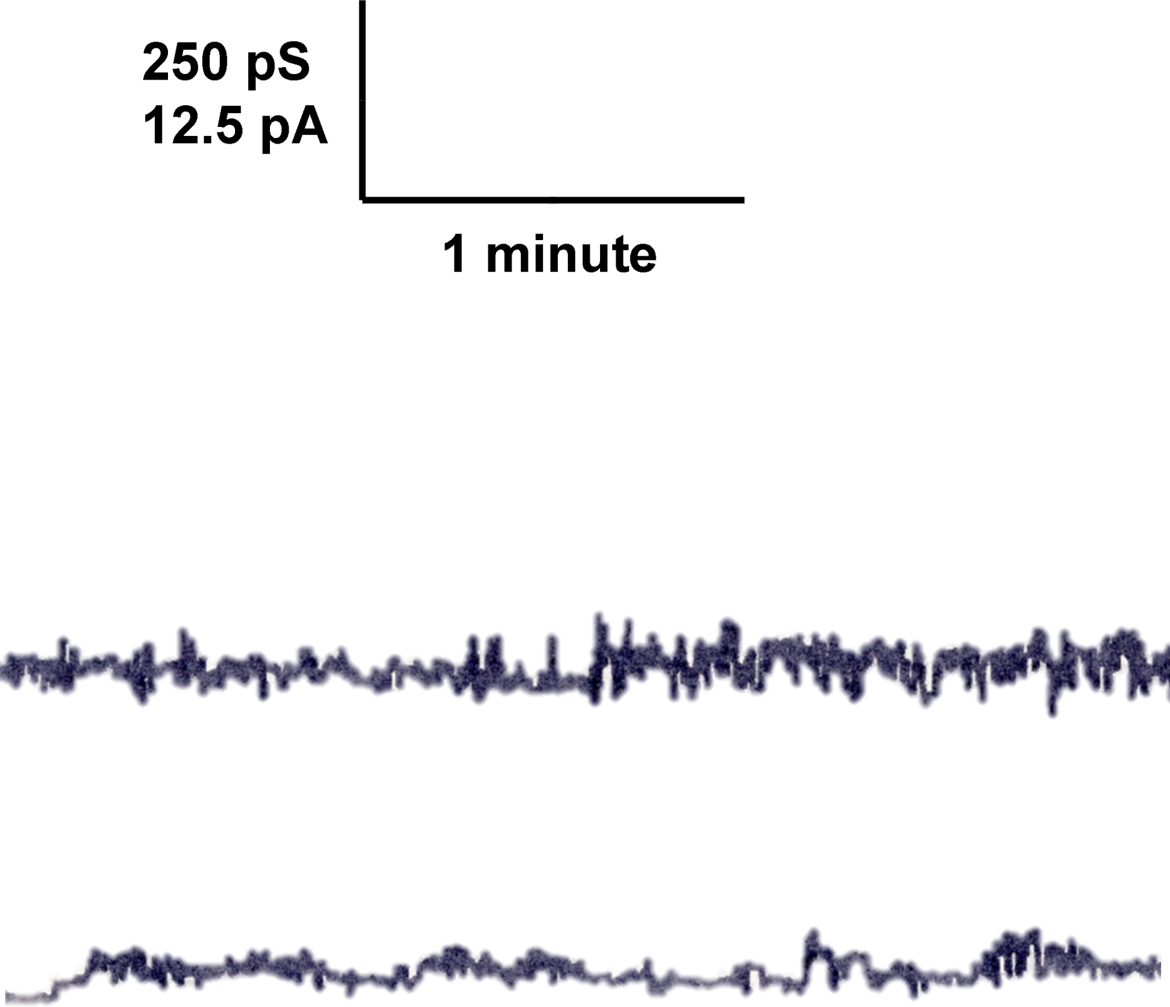
Single-channel recording of a PC/*n*-decane membrane in the presence of enriched outer membranes of *C*. *crescentus* ΔompW mutant. The aqueous phase contained 1 M KCl and 100 ng ml^−1^ protein from enriched outer membranes treated with 0.5% LDAO. The applied membrane potential was 50 mV; *T* = 20°C. Note that the recording starts at the lower trace about 2 minutes after addition of the membrane extract and then continues in the upper trace.

The lipid bilayer method is very sensitive for the presence of membrane-active material, such as minor contaminants of gram-negative bacterial porins. Although it is relatively unlikely that an impurity in the purified *C*. *crescentus* OmpW was responsible for the channels observed in lipid bilayer membranes, we could not completely exclude such a possibility. Therefore *ompW* was knocked out in the genome of *C*. *crescentus* by crossing out *ompW* using the pKMOBsacB-ompW-A/B plasmid (see Materials and Methods). Fig 5 shows an SDS-PAGE of PBS-EDTA extractions from the wildtype strain stained with Coomassie (lane 1) and *C*. *crescentus* CB15 NA1000 353Φ (JS1013) Δ*ompW* (lane 2). OmpW present in wildtype *C*. *crescentus* (lane 1; indicated by a star) is clearly absent in the mutant strain (lane 2). Lipid bilayer experiments were performed with enriched outer membrane extracts of the *C*. *crescentus* OmpW-deficient mutant extracted with 0.5% LDAO. When small amounts of this detergent extract were added to one or both sides of a black PC/n-decane membrane we observed only insignificant effects on membrane conductance and no conductance steps of the type shown in Figs 1 and 3 (see Fig 6).

## Discussion

### The enriched outer membrane of *C*. *crescentus* contains a cation-permeable channel

Here we demonstrated the presence of a channel in the enriched outer membranes of the alpha proteobacterium *C*. *crescentus*. This was quite surprising because its genome does not contain genes that code for the classical outer membrane porins [13]. Nevertheless, it is clear from this study that *C*. *crescentus* also contains at least one outer membrane channel, which was definitely absent in the OmpW deficient *C*. *crescentus* strain. The 20 kDa band excised from preparative SDS-PAGE of enriched OM of *C*. *crescentus* wildtype had a very high channel-forming activity. No other protein bands excised from gels showed channel-forming activity. Similarly, no channel-forming activity was found in outer membrane extracts of the OmpW-deficient *C*. *crescentus* mutant suggesting that the outer membrane of *C*. *crescentus* contains this one porin-like channel. The channel had a single-channel conductance of about 120 pS in 1 M KCl. The channel-forming protein was subjected to partial sequencing and was identified as OmpW of *C*. *crescentus*. The OmpW porin family is widespread amongst Gram-negative bacteria, but with no well established function, perhaps with the exception of the transport of quaternary ammonium compounds (QAC), and methyl viologen dichloride (MV) transport by OmpW of *Escherichia coli* [44]. The channel functions of OmpW of *E*. *coli* and OprG of *P*. *aeruginosa* were postulated to be involved in the uptake of hydrophobic compounds [18, 19]. It has been suggested that the channel function of OmpW of *E*. *coli* and OprG of *P*. *aeruginosa* may be plugged by W155 and W170, respectively (see below) [17–19].

It is noteworthy, that the genome of *C*. *crescentus* also contains the gene coding for a second OmpW-like protein (CC_1287). CC_1287 is only distantly related to OmpW (CC_1409) because from a total of 206 amino acids of the mature protein only 63 amino acids (30.6%) are identical. We found no indication that this protein is expressed in appreciable amounts in wildtype *C*. *crescentus* or in the OmpW-deficient mutant. This means that the properties of this OmpW-like protein did not interfere with the results of OmpW (CC_1409) described here and need to be studied in future.

### Properties of OmpW of *C*. *crescentus* outer membrane

**Fig 7:**
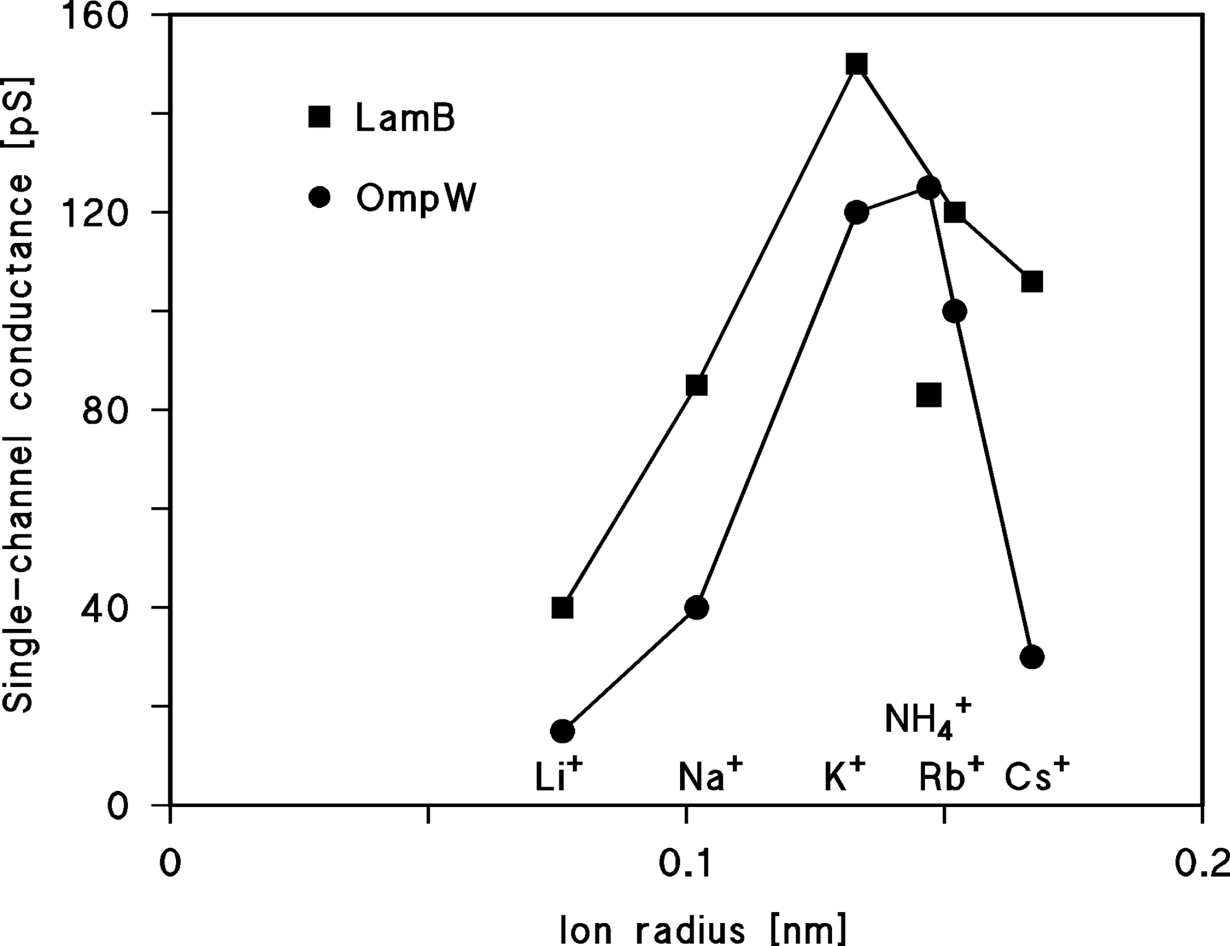
Single-channel conductance of OmpW in 1 M salt solution as a function of the ionic radii of monovalent cations. The data were taken from Table 1. The single-channel conductance of LamB of *E*. *coli* is given for comparison [45].

OmpW of *C*. *crescentus* was found to be highly cation-selective. Its selectivity for potassium ions over chloride was at least 10-fold; the calculated value was about 15. However, it has to be kept in mind that the permeability ratio P_a_/P_c_ reacts in this range very sensitive to small changes of the zero-current membrane potential [9]. This means that we found very little indication for the permeation of anions through OmpW because the single channel conductance in 1 M KCl was the same as in 1 M potassium acetate, despite the fact that chloride in the aqueous phase has a much higher mobility than acetate. In addition, the single channel conductance was not a linear function of the bulk aqueous salt concentration (see Table 1). Instead, the conductance showed saturation because the single-channel conductance increased from 10 mM to 3 M KCl only by a factor of 5 despite a change of the salt concentration by a factor of 300. Experiments with different alkaline cations indicated clearly that OmpW does not form a wide water-filled pore, which would be typical for most Gram-negative and Gram-positive bacterial porins, even if they contained point charges [3, 43]. Instead, OmpW of *C*. *crescentus* appeared to be an ion channel; its single channel conductance had a maximum for ammonium and potassium ions and became much smaller for bigger cations indicating that they likely loose part of their hydration shell while moving through the channel. This argues for a small negatively charged selectivity filter inside the channel. Presumably, the ionic radii of the different cations, and not the sizes of their hydration shells, play an important role for their permeation through OmpW. Fig 7 shows the single channel conductance of OmpW as a function of the ionic radii of the ions [38–40]. The conductance data for another small substrate-specific and cation-selective channel (LamB of *E*. *coli*) is given for comparison [45]. The relationship for OmpW follows approximately the Eisenman series VI for carrier-mediated transport of cations or for the transport of monovalent cations through channels [46]. This suggests that the field strength inside the channel is presumably medium sized. Ion transport trough general diffusion channels of enteric bacteria follow instead Eisenman series I or II, indicating very low field strength in the channels [46]. The minimum diameter of the OmpW channel is presumably close to that of Cs^+^ and smaller than that of N(CH_3_)_4_^+^ ions because larger organic cations have a very low, if any, permeability through OmpW. Table 1 shows also that the crystal radius of certain cations is larger that the hydrated ion (Stokes’ radius), contrary to expectation. This is a well-known phenomenon, which is presumably caused by some in theory neglected interaction of ions with the water molecules [42].

OmpW of *C*. *crescentus* functions as a cation-permeable channel, having a certain preference for divalent cations, since calcium and magnesium ions had a higher permeability through OmpW than potassium ions. Preference for other solutes was not found. This could indeed mean that it is a channel for the transport of cations. It is noteworthy that such a function has not previously been established for OmpW of other bacteria but it has been suggested that OmpW is involved in export of methyl viologen dichloride (MV) in combination with the small multidrug resistance (SMR) transporter protein EmrE [44]. The reason for this is that OmpW is a rather small channel with only eight beta-strands and has little possibility for the passage of solutes, such as larger quaternary ammonium compounds (see Table 1). Previous data from structural studies of OmpW of *E*. *coli* and OprG of *P*. *aeruginosa* suggested that members of the OmpW family could be involved in the transport of small hydrophobic molecules across the bacterial outer membrane [18, 19]. Lipid bilayer experiments with OmpW of *E*. *coli* suggested that it forms small ion-permeable channels with a conductance of about 20 pS in 1 M KCl [18], which is considerably lower than that of *C*. *crescentus* OmpW reported here. The 3-D structure of *Pseudomonas aeruginosa* OprG, another member of the OmpW family, was resolved at 2.4 Å resolution [19]. Again, the structure suggested that OprG forms a channel for the diffusion of small hydrophobic molecules, although lipid bilayer experiments proposed a single-channel conductance of about 500 pS for OprG, which is relatively high if the narrow size of the channel’s 3-D structure is considered [47]. This means that OmpW of *C*. *crescentus* forms a channel that has, despite sequence homologies with OmpW of *E*. *coli* and OprG of *P*. *aeruginosa* (see below), a completely different function than structurally related outer membrane proteins.

### Model of OmpW of *C*. *crescentus*

**Fig 8:**
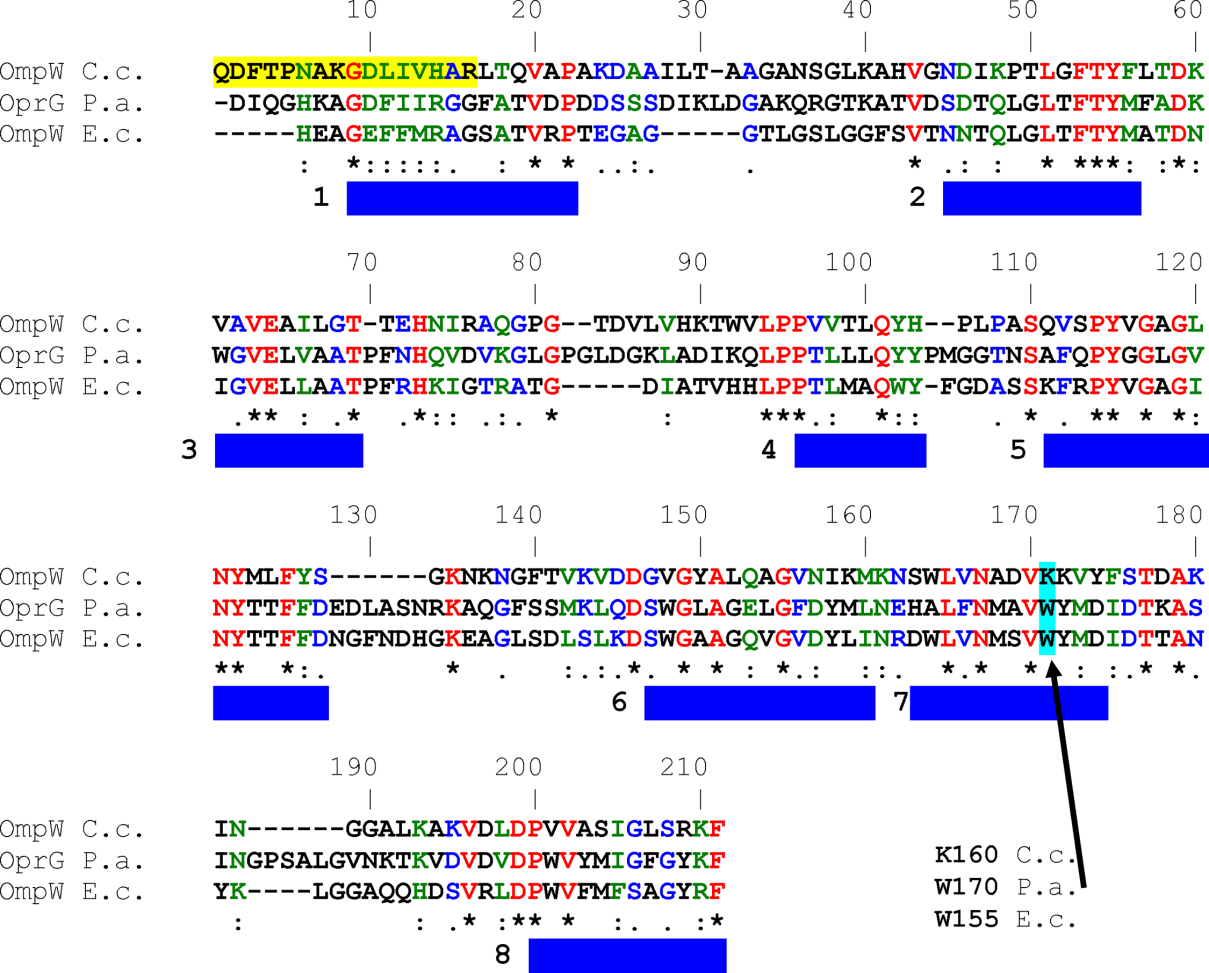
Amino acid sequence alignment of OprG of *P*. *aeruginosa*, OmpW of *E*. *coli* and OmpW of *C*. *crescentus*. The alignment was performed using Pole Bioinformatique Lyonnaise Network Protein Sequence Analysis (http://npsa-pbil.ibcp.fr). Amino acids identical in all three proteins are highlighted in red, strongly similar amino acids (:) are given in green and weakly similar ones (.) in blue. The replacement of W155 of OmpW of *E*. *coli* and W170 of OprG of *P*. *aeruginosa* by K160 of *C*. *crescentus* is given in green color and is indicated by an arrow. The eight beta strands in OprG of *P*. *aeruginosa* and in OmpW of *E*. *coli* are numbered and indicated by blue bars [18, 19]. The yellow highlighted sequence was found by mass spectrometric analysis of tryptic peptides using Mascot N-terminal sequencing of OmpW of *C*. *crescentus* (http://www.matrixscience.com/).

Sequence comparison of OmpW of *C*. *crescentus* with other members of the OmpW family suggested that it had highest homology with OmpW of *Caulobacter segnis* ATCC 21756, *Asticcacaulis excentricus* CB 48, *Brevundimonas* sp. BAL3 and *Phenylobacterium zucineum* HLK1 (Basic Local Alignment Search Tool, [34,35]). These bacteria are closely related to the genus *Caulobacter*, belonging to the family *Caulobacteraceae*, order *Caulobacterales*. All these OmpW polypeptides are similar in length to *C*. *crescentus* OmpW (200 to 250 amino acids) and share many strategically positioned conserved residues. The homology of the *C*. *crescentus* OmpW to the OmpW proteins with known 3D-structure (OmpW of *E*. *coli* and OprG of *P*. *aeruginosa*) is less pronounced, but still obvious (amino acid identity is approximately 32% in both cases and 19.4% for all three proteins (Pôle BioInformatique Lyonnais) [18,19]). 41 amino acids, including many glycines and several prolines, appear to be highly conserved between all three OmpW family proteins. This allows a meaningful comparison between the three different OmpW species (see Fig 8) and it is also possible to build the putative structural model of OmpW of *C*. *crescentus* using homology modeling approach [32] (see below).

**Fig 9:**
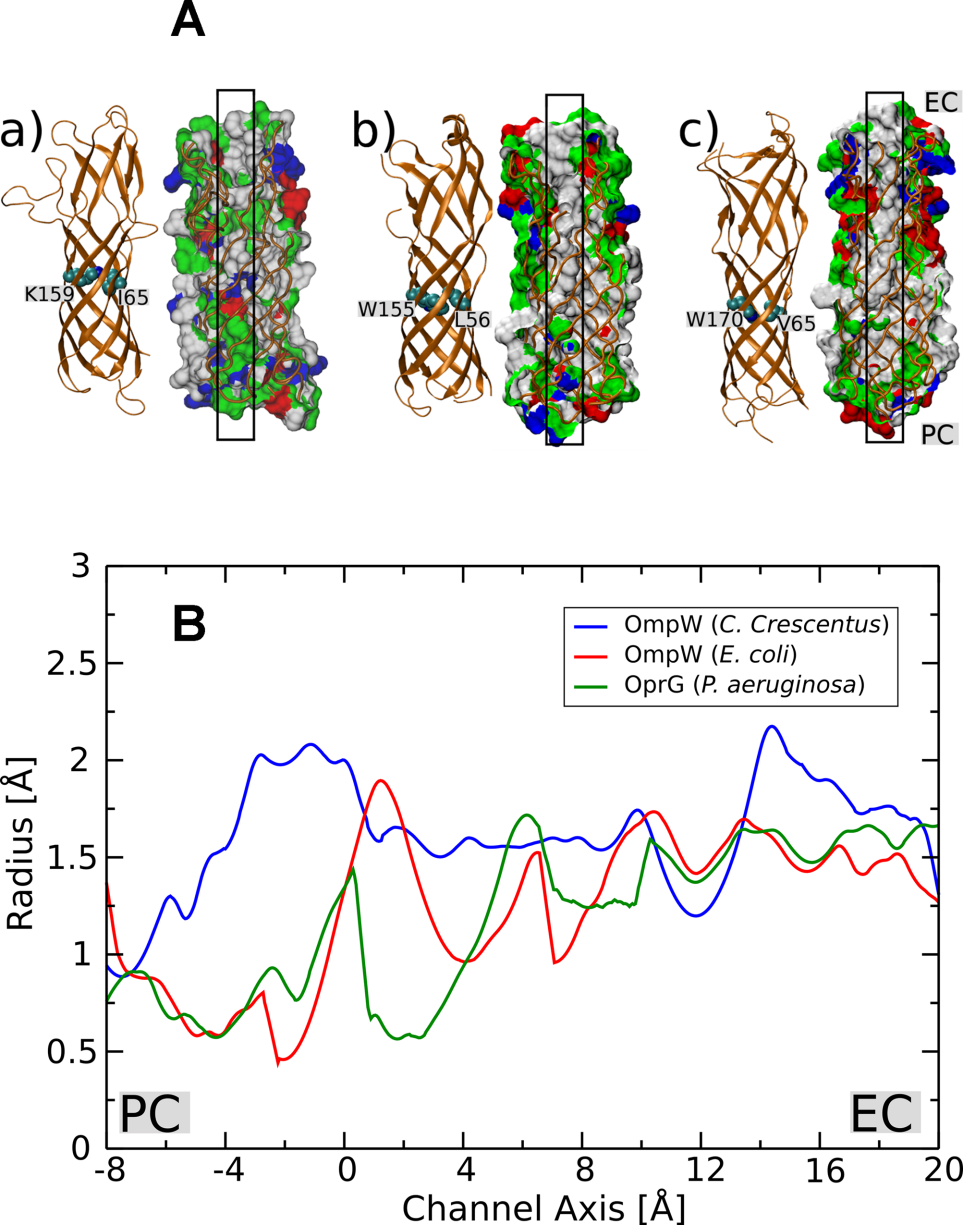
Structural properties of OmpW of *C*. *crescentus*. A. Comparison of structural features between a) OmpW *C*. *crescentus*, b) OmpW *E*. *coli* and c) OprG *P*. *aeruginosa*. Residues that are a part of a putative hydrophobic gate in *E*. *coli* (W155 and L56) and *P*. *aeruginosa* (W170 and V65) channels are shown as spheres. The corresponding residues in OmpW *C*. *crescentus* (K159 and I65) are also shown. Moreover, all channels are shown using a surface representation and color coded according to the residue type (Green: hydrophilic, white: hydrophobic, red: acidic, blue: basic). The channels are cut from the front to enable visualization of the channel surface interior. The black colored box indicates a putative ion transport pathway across the channel. EC and PC denote the extracellular and periplasmic sides of the channel, respectively. The models of the three OmpW homologs were modeled using the program Modeller [32], taking the structure of *E*. *coli* OmpW as a template [18]. B. Comparison of channel radii between *C*. *crescentus* OmpW (blue), E. coli OmpW (red) and OprG of *P*. *aeruginosa* (green) along the channel axis. The channel radii were calculated using the program HOLE [51].

The small and hydrophobic *E*. *coli* OmpW channel has a conductance of approximately 20 pS in 1 M KCl [18]. In contrast, *C*. *crescentus* OmpW formed a channel with a relatively high conductance of 120 pS in 1 M KCl in our bilayer measurements. Comparison between the model of OmpW and the known structures of these OmpW family proteins revealed possible factors that could contribute to a larger conductance in *C*. *crescentus* OmpW. The crystal structures suggested that both *E*. *coli* OmpW and *P*. *aeruginosa* OprG possess a hydrophobic gate in the central region of the channel (Fig 9A; b and c). Both channels have a tryptophan residue as a part of the hydrophobic gate, which possibly makes the passage of ions very difficult. The tryptophan residues (W155 of OmpW of *E*. *coli* [18] and W170 of OprG of *P*. *aeruginosa* [19]), are replaced by K160 in OmpW of *C*. *crescentus*, (Fig 9A; a) which is unlikely to hinder passage of ions through the channel. In addition, *C*. *crescentus* OmpW has a channel interior with higher hydrophilicity (Fig 9A; a, as shown by green color surface within the black box) when compared to *E*. *coli* OmpW (Fig 9A; b) and *P*. *aeruginosa* OprG (Fig 9A; c), which indicates that this channel has a function different from that of the homologues of *E*. *coli* and *P*. *aeruginosa*. The interior surfaces of the *E*. *coli* OmpW and *P*. *aeruginosa* OprG channels are very hydrophobic (white color surface) and possibly make the permeation of ions through the channel energetically unfavorable. *C*. *crescentus* OmpW, on the other hand, due to its relative hydrophilic environment, may not provide such a permeation barrier to ion passage and consequently has relatively higher conductance.

Moreover, we examined the contribution of the pore size towards higher conductance of *C*. *crescentus* OmpW. To this end, a slightly larger radius of *C*. *crescentus* OmpW channel was observed compared to those of the other two channels in several regions, particularly from the center of the channel towards the periplasmic region (Fig 9B). Another important feature of the OmpW family channels from *E*. *coli* and *P*. *aeruginosa* is the presence of a lateral opening in the barrel wall, which is suggested to allow diffusion of small hydrophobic solutes across the outer membrane by a lateral diffusion mechanism [18, 19]. In our model of *C*. *crescentus* OmpW, we do not observe such an opening in the channel although we cannot completely exclude such a possibility. This further supports the view that the *C*. *crescentus* channel may have a different function from that of *E*. *coli* OmpW and *P*. *aeruginosa* OprG. On the other hand, only high-resolution data such as X-ray protein crystallography may support the possibly different function of OmpW of *C*. *crescentus* as compared to the other OmpW-homologs. Interestingly, the structure of OprG shows additional short beta-strands (not shown in Fig 9) that are not membrane spanning. These beta-strands seem to extend over the thickness of the outer membrane, which means that they form long external loops [18, 19]. Here we have provided clear evidence that OmpW could be responsible for the uptake of cations in *C*. *crescentus*.

*C*. *crescentus* has a requirement for calcium, though it is not clear where the absolute requirement lies. Calcium ions are needed for assembly of the crystalline S-layer protein RsaA [48]. Since RsaA is secreted by a type I secretion mechanism, it is likely that calcium is also needed for either secretion or folding of the protein following secretion, in a manner analogous to all other type I secreted proteins. However, the S-layer is a completely dispensable structure. Moreover, the type 1 secretion apparatus spans both the cytoplasmic and outer membranes; hence for S-layer biogenesis there is no apparent need for a channel that enables cation transport to the periplasm [49]. There are, however, additional still undefined roles for calcium ions. Mutants no longer requiring calcium for growth can be isolated [50]. One consequence of all these mutants is the loss of the O-side chain of lipopolysaccharide that is used for S-layer surface attachment. Therefore, these so-called calcium-independent mutants all shed RsaA into the culture medium. This causal relationship has not been deciphered, but since O-side chain biosynthesis involves synthesis activities within the periplasmic space, it may be that a calcium-selective OmpW porin plays a specific role in this process.
